# Demystifying non-coding GWAS variants: an overview of computational tools and methods

**DOI:** 10.1093/hmg/ddac198

**Published:** 2022-08-16

**Authors:** Marijn Schipper, Danielle Posthuma

**Affiliations:** Department of Complex Trait Genetics, Center for Neurogenomics and Cognitive Research, Amsterdam Neuroscience, VU University Amsterdam, De Boelelaan 1105, Amsterdam 1081HV, The Netherlands; Department of Complex Trait Genetics, Center for Neurogenomics and Cognitive Research, Amsterdam Neuroscience, VU University Amsterdam, De Boelelaan 1105, Amsterdam 1081HV, The Netherlands

## Abstract

Genome-wide association studies (GWAS) have found the majority of disease-associated variants to be non-coding. Major efforts into the charting of the non-coding regulatory landscapes have allowed for the development of tools and methods which aim to aid in the identification of causal variants and their mechanism of action. In this review, we give an overview of current tools and methods for the analysis of non-coding GWAS variants in disease. We provide a workflow that allows for the accumulation of *in silico* evidence to generate novel hypotheses on mechanisms underlying disease and prioritize targets for follow-up study using non-coding GWAS variants. Lastly, we discuss the need for comprehensive benchmarks and novel tools for the analysis of non-coding variants.

## Introduction

Charting the regulatory function of the non-coding genome has been an ongoing effort for over a decade. Although the claims of the ENCODE project that 80% of the whole genome is functional is heavily debated (1,2), a plethora of regulatory features, ranging from small binding motifs to changes in chromatin-packing, has been discovered in what was once considered to be ‘junk’ DNA (3,4). These mechanisms reveal a strong involvement of the non-coding genome in gene regulation (5).

The role of protein-coding variants in disease is relatively clear. Loss or gain of function mutations can disrupt normal protein function and are therefore able to exert potentially detrimental effects on a phenotype (6). The role of a non-coding variant is less obvious. Genome-wide association studies (GWAS) have found an abundance of statistical associations between both coding and non-coding variants and disease. How these associated variants may impact biological functions, provides insight into the genetic background of disease susceptibility. When considering fine-mapped GWAS hits, a strong enrichment in coding variants and a small depletion of non-coding variants are observed when compared with the expected distribution of GWAS hits given the size of the coding and non-coding genome (7). This indicates that a given coding variant is more likely to be statistically associated with phenotypical change than a non-coding variant. Nevertheless, since vast majority of the genome is non-coding, we observe that approximately 95% of the high confidence fine-mapped SNPs are in non-coding and flanking regions (7). This implicates a substantial role for non-coding variation in disease.

Research into the regulatory functions of non-coding DNA has allowed for the development of a host of computational tools that aid in the interpretation of disease-associated non-coding variants. Here we provide a non-exhaustive overview of current tools and methods which can be used to interpret and generate hypotheses on the role of non-coding disease-associated variants identified by GWAS.

## Exploring GWAS results in an era of data abundance

As the amount of performed GWAS has seen a steady increase, so has the number of available resources at a researcher’s disposal to interpret GWAS results. This is particularly helpful for analyzing non-coding variants, where there are no obvious protein alterations that may explain phenotypic effects. Instead, these non-coding variants impact the phenotype by alteration of regulatory elements such as enhancers (8,9), transcription factor binding sites (10) and chromatin state (11). To give an example in a disease context: multiple studies have linked point mutations in the promotor sequence of the *TERT* gene to cancer (12–15). Relevant resources for the regulatory functions of non-coding regions can be found in the ENCODE (3,16), FANTOM5 (17), Epigenomics Roadmap (18) and GTEx consortium atlas (19) projects. Yet the abundance of information available from these resources renders manual annotation of variants of interest time-consuming, inefficient and error-prone.

To address this problem, specific tools have been developed that automatically annotate variants, and cross-reference them with relevant data repositories. For example, ANNOVAR (20), FUMA (21), GEMINI (22), HaploREG (23), RegulomeDB (24) and VEP (25) annotate variants with a broad range of sources, including the resources mentioned before (Table 1). The GWAS results can be visually explored using LocusZoom (26) or FUMA (21). FUMA annotates and visualizes GWAS risk loci and allows for interactive investigation of GWAS results. LocusZoom visualizes the Manhattan plots of risk loci and their underlying linkage-disequilibrium structure. FUMA provides a demonstration case of the relevancy of these annotation tools in investigating non-coding variants in disease: several non-coding variants associated with BMI are located in an intronic region of the *FTO* gene, which was thought to be the causal genes. Annotation of these variants revealed them to be expression quantitative trait loci (eQTLs) for *IRX3*, which functional studies revealed to be the actual causal gene (27), exemplifying the use of variant annotation tools in non-coding variant analysis (21). Most of these tools were not developed exclusively for the exploration of non-coding variants, yet they are a useful first step in the analysis of disease-associated non-coding variants.

**Table 1:** Commonly used annotation, exploration and visualization tools of possible (coding and) regulatory functions of SNPs

Name	Description	Strengths and weaknesses	Non-coding specific
ANNOVAR (20)	Automatic annotation of variants	+ Integrates a large number of prediction tools + Additional annotation databases downloadable − Requires affinity with command line	No
HaploREG (23)	Automatic annotation of variants with non-coding functional studies	+ Non-coding specific + User-friendly web portal	Yes
RegulomeDB (24)	Automatic annotation of variants with non-coding functional studies	+ Non-coding specific + User-friendly web portal	Yes
GEMINI (22)	Automatic annotation of variants	+ Flexible querying of relevant databases + Allows for integration of custom annotations − Affinity with command line tools needed	No
GLANET (68)	Automatic annotation of variants	+ Includes enrichment analysis of genomic elements + User-friendly GUI	No
Jannovar (69)	Automatic annotation of variants	+ User-friendly Java-based GUI + Includes API for automated annotation	
SnpEff (70)	Automatic annotation of variants and variant effect prediction	+ User-friendly analysis toolbox + Easy to integrate with tools such as GATK and Galaxy − Results vary per tool	No
VARAdb (71)	Automatic annotation of variants	+ User-friendly web portal + Allows for easy prioritization per score − Only contains variants found significant for a disease GWAS − Max 100 variants	No
VEP (25)	Automatic annotation of variants and variant effect prediction	+ Plugins allow for non-coding variant effect predictors to be integrated into the analysis − No built-in non-coding variant prioritization	No
LocusZoom (26)	Visualization of risk loci	+ User-friendly web portal − No in-depth annotation	No
FUMA (21)	Annotation and visualization of GWAS results	+ User-friendly web portal + Broad range of analyses + Interactive visualizations − No batch submissions possible − Cannot add custom annotation data to analysis	No

## Predicting non-coding variant effects

When interpreting common or rare variants in a disease risk locus, it is often unclear which non-coding variants may have phenotypical consequences. Although the tools mentioned in the previous paragraph help annotate data with a range of functional genomic features, it can still be difficult to identify whether a polymorphism within a feature will have a phenotypic effect. For example, if ANNOVAR annotates a variant to be within an enhancer region, this does not indicate that the variant alters the enhancer function. To this extent a range of tools has been developed which aim to infer the effect of inputted variants (Table 2). These tools aim to predict whether a variant has a functional effect or is pathogenic based on the local genetic sequence and features. These predictions are mostly made outside of a specific context, such as cell type or disease. In other words, a variant predicted to be pathogenic/functional will most likely have a phenotypical effect but it is unknown what the precise effect may be on your trait of interest. Nevertheless, these tools can be useful for identifying potential causal variants from a list of disease-associated variants for further investigation.

**Table 2:** Commonly used variant effect prediction tools

Name	Description	Method	Strengths and weaknesses	Non-coding specific
ARVIN (72)	Random forest classifier leveraging disease-specific gene regulatory networks	Supervised machine learning	+ Disease-specific − Affinity with R needed	Yes
CADD (35)	SVM trained on distinguishing alleles that are fixed in the human population vs simulated *de novo* variants using 63 predictive annotation features	Support vector machine (SVM)	+ Web portal + Precomputed scores for reference variants − Open-ended scoring scheme makes for non-interpretable final scores	No
DANN (36)	A deep neural network developed using the CADD training data	Deep neural network (DNN)	+ Precomputed scores for reference variants − Some Python/command line affinity needed	No
DeepSEA (38)	A deep neural network trained on sequence context of ENCODE and Roadmap Epigenomic elements and epigenomic profiles	Deep neural network (DNN)	+ Web portal + Allows for sequence analysis (FASTA) + Benchmarked as performing well across a variety of datasets	Yes
DeltaSVM (39)	Gapped *k-*mer support vector machines trained on DNA sequencing encoding cell-type specific regulatory elements	Gapped *k*-mer support vector machine (gkmSVM)	+ Cell type-specific − Command line or R affinity needed	Yes
DIVAN (33)	Ensemble learner trained per disease on multiple genomic features and epigenetic profiles. DIVAN aims to reduce noise by selecting disease-relevant features only	Supervised machine learning	+/− Precomputed scores available for 45 diseases − Some R/command line affinity needed	Yes
EIGEN (29)	Spectral meta-learner trained on annotations of 1000 genomes variants not present in dbNSFP and maximally 500 upstream of TSS. Eigen assumes that the annotations for a given set of variants naturally separate into two distinct groups: functional and non-functional. EIGEN assigns meta-scores to each variant which indicate whether the variant is predicted to be part of the functional or non-functional group	Unsupervised machine learning	+ Precomputed scores available for reference genome − Some R affinity needed	No
FATHMM-MKL (31)	Multiple kernel learning framework trained on annotations of germline HGMD variants	Supervised machine learning	+ Web portal + Benchmarked as best performing for rare germline variants − Score not interpretable	No
FATHMM-XF (32)	Multiple kernel learning framework trained on annotations of germline HGMD variants. Expanded features from FATHMM-MKL	Supervised machine learning	+ Web portal + Benchmarked as best performing for rare germline variants − Score not interpretable	No
FunSeq2 (34)	Informative features of genomic context were created by comparing functional data with 1000 genomes, ENCODE, COSMIC and CGC pathogenic variants. Variants’ pathogenicity is predicted using a weighted scoring scheme of the created genomic contexts	Weighted scoring framework	+/− Specialized in finding cancer-related pathogenic variants + Web portal + Benchmarked as performing well across a variety of datasets	Yes
GWAVA (30)	Random forest classifier trained on variants in the HGMD. GWAVA uses nine main types of features for prediction (e.g. genomic context, open chromatin and TF binding)	Supervised machine learning	+ Web portal + Precomputed scores available for reference genome	Yes
JARVIS (37)	Convolution neural network and deep neural network trained on WGS constraint scores, genomic sequence and functional annotations	Supervised machine learning	+ Precomputed scores available for reference genome − Python affinity needed	No
LINSIGHT (28)	A linear model of multiple genomic features fitted on human and primate conservation data	Multiple regression	+ Benchmarked as performing well across a variety of datasets	Yes

The host of variant pathogenicity prediction tools makes use of different strategies and functional data. Current methods commonly apply one or more of the following three strategies (28):

(1) Machine learning classification relying on the integration of genomic annotation features (EIGEN (29), GWAVA (30), FATHMM-MKL (31), FATHHM-XF (32), DIVAN (33) and FunSeq2 (34)).

(2) Leverage evolutionary data, combined with functional genomic data to predict variant deleteriousness (CADD (35), DANN (36), JARVIS (37) and LINSIGHT (28)).

(3) Motif disruption-based prediction, where the disruption of genetic features such as gain/loss of transcription factor binding sites or splicing sites indicate pathogenicity (DeepSEA (38), DeltaSVM (39) and JARVIS (37)).

The diversity of methods attests to the competitiveness in pathogenicity prediction algorithm development. With the large variety of tools, utilizing different methods and background data, it can be difficult to select one that best suits a given research project.

Benchmarking this variety of predictive tools is not a trivial task. Typically, a small proportion of tools is chosen to predict variant pathogenicity and benchmarked based on one or two reference databases (e.g. ClinVar or COSMIC). In a recent benchmarking of 24 pathogenicity prediction tools, Wang *et al*. showed that there is a large variation in tool performance depending on the chosen benchmarking dataset. In this benchmark, four ground truth datasets were constructed using rare somatic cancer variants from COSMIC, rare germline variants from ClinVar, regulatory variants from multiple eQTL databases and disease-associated GWAS variants. LINSIGHT and FunSeq2 were the best performers across all four benchmarking datasets (40). Where FATHMM-MKL was the clear winner in a previous benchmark (41), the newer FATHMM-XF was the top performer only on the ClinVar benchmarking set. The earlier benchmark also reports higher predictive accuracy by GWAVA for variants in the COSMIC database than any of the 24 tools benchmarked by Wang *et al*. (41). It is relatively unclear how the motif-focused deep learning tools JARVIS and DeepSEA perform compared with other tools across different datasets. From this brief overview of recent benchmarking efforts of pathogenicity prediction algorithms, it is evident that none of the tools consistently outperform all others across every benchmarked reference dataset, suggesting that each tool has different sensitivities to the underlying genetic architecture that is most represented in each reference database. Cooper *et al*. also demonstrate that variant pathogenicity prediction methods correlate poorly with each other and further show that they also correlate poorly with the results from massive parallel reporter assays for non-coding variants associated with Alzheimer’s disease and Progressive Supranuclear Palsy. This implies the need for experimental validation after prioritization using pathogenicity prediction tools (42).

Despite their shortcomings, these tools have proven useful for the prioritization of causal disease variants for follow-up studies. Examples of their practicality in disease research are many. FATHMM and LINSIGHT have proven useful in pinpointing novel functional mutations in *PMS2* in cancer genomes (43). CADD has aided in prioritizing pathogenic variants associated with Alzheimer’s disease (44). Given the large variation in performance of the currently available tools, it is clear that when it comes to a one-size-fits-all pathogenicity prediction tool we are still far from the end-game. As such, the 2013 clinical guidelines for categorizing a non-coding variant as pathogenic, which decree the use of at least three different computational tools, still seem relevant today (45). When selecting algorithms, we encourage the consultation of recent benchmarks on a dataset containing variants with an expected similar genetic architecture as the input variants for the best performing tools. Overall, when combined and selected with care, pathogenicity prediction algorithms are a powerful tool for extracting potentially causal variants from a list of variants of interest.

## Quantitative trait loci: a black box approach

One of the core difficulties in non-coding variant analysis is the identification of the mechanisms and biological pathways through which a variant might impact a phenotype. Linking variants to effector genes allows us to shift the analysis to a more interpretable unit of study. One approach to link variants to genes is colocalizing variants with molecular quantitative trait loci (molQTL). MolQTLs are associations between the presence of a variant and a molecular measurement, such as RNA expression levels (eQTL), protein abundance (pQTL) or differential splicing (sQTL). Although molQTLs are blind to the precise mechanism of action, they provide a direct link from variant to gene. To incorporate this data for the analysis of non-coding variants, colocalization methods, such as COLOC (46) and ezQTL (47), have been developed to test whether the overlap between the GWAS and molQTL signal is statistically significant (Table 3). A different molQTL approach has been developed in the machine learning classifiers FIRE (48) and TIVAN (49) (Table 3). Both methods embody machine learning classifiers trained on annotated cis-regulatory eQTL variants, which aim to predict whether input variants are QTLs for nearby genes, which can aid causal variant interpretation. Note that these tools were not trained to predict trans effects which might arise from chromatin interaction for example. MolQTL analyses have increased the interpretability of non-coding variant risk alleles of diseases such as type 1 diabetes (T1D) and schizophrenia. Through eQTL analysis, it was found that two T1D risk alleles converge in upregulating interferon-γ response genes (50). Dysregulation of the genes *FURIN*, *TSNARE1*, *CNTN4*, *CLCN3* and *SNAP91* have been implicated by eQTL analysis of schizophrenia GWAS hits (51), which were later separately prioritized by chromatin interaction analysis of schizophrenia risk variants (52).

**Table 3:** Commonly used QTL analysis tools

Name	Method	Description	Strengths and weaknesses	Non-coding specific
COLOC (46)	Bayesian testing framework	COLOC allows for the assessment of whether GWAS signals are owing to the same causal variant	+ Interpretable results − Some affinity with R needed − Loses power when multiple causal variants present at the locus	No
eCAVIAR (73)	Probabilistic model	eCAVIAR fine-maps and colocalizes GWAS signals from one or more causal variants with eQTLs	+ Interpretable results + Retains power when multiple causal variants are present − Accurate WGS reference panel needed for fine-mapping − affinity with command line tools needed	No
ezQTL (47)	ezQTL	Performs colocalization analysis and visualizes QTL results	+ User friendly + Automated visualization	No
fastENLOC (74)	Bayesian hierarchical colocalization	fastENLOC allows for post-fine-mapping colocalization analysis. FastEnloc automatically parameterizes the enrichment of eQTLs in the GWAS	+ Works well for strongly QTL enriched or depleted GWAS traits + Retains power when multiple causal variants are present − affinity with command line tools needed —performs best on fine-mapped data	No
FIRE (48)	Random Forest	FIRE is a Random Forest classifier trained to predict whether a variant is a cis-eQTL for nearby genes	+ Pre-computed FIRE scores available − Does not predict for which gene variant is an eQTL	No
LocusCompare (75)	FINEMAP and eCAVIAR	Platform with readily available visualizations of colocalization of 200 GWAS	+ Easy to use web portal − Users are not able to upload their own summary statistics	No
TIVAN (49)	Ensemble learning	TIVAN is an ensemble classifier that aims to predict whether a variant is a cis-eQTL for nearby genes in specific tissues	+ Tissue specific analysis − Some affinity with R needed − Does not predict for which gene a variant is an eQTL when multiple genes are close to variant	No

Despite their practicality, QTLs are far from perfect. One should be aware that the molQTLs can be tissue or cell-type specific. If the investigated trait of interest is analyzed based on molQTLs of non-relevant tissues or cell types this could lead to incorrect prioritization of genes (53). Preliminary results from a recent investigation show GWAS hits and eQTLs have a different makeup of genomic features, suggesting that a large part of GWAS hits cannot be explained by eQTLs (54). Still, QTLs can be a powerful tool for interpreting the role of non-coding variants in disease when appropriate tissue types from well-powered samples are combined with additional lines of evidence.

## Linking variants to genes and regulatory mechanisms

Most of the earlier mentioned methods pool a variety of genomic annotations and features to make *in silico* predictions of pathogenicity or prioritize genes. It can therefore be hard to interpret why a specific variant is predicted to be pathogenic in a specific disease context. Linking variants to genes or epigenetic effects allows for easier integration with known information on the studied disorder. Hi-C (55), enhancer studies (17,56,57), chromatin accessibility (58,59), DNase I hypersensitivity site–gene promotor correlation (60) and the activity-by-contact (61) model can all be used to connect the genomic region containing the variant of interest to a gene it potentially regulates. This does not immediately implicate that the variant alters normal gene regulation. Recently, massive parallel reporter (42,62) and CRISPR/Cas9 (63) have been used to assess whether variants in a single genomic position alter gene regulation. Using data related to a single (epi)genetic mechanism to link non-coding variants to genes allow for easier interpretation than molQTLs, and provide more concrete evidence of biological functionality of the variant of interest than *in silico* pathogenicity predictions.

The downside of using functional data for prioritizing potentially causal genes in a non-coding locus is that these datasets might not cover the relevant tissue or cell type and that different datasets/strategies often indicate different causal genes (72). This makes integrating multiple data sources for the prediction of the causal gene for a GWAS signal difficult. Recent attempts to solve this integration problem through the use of machine learning and regression models which predict causal genes from coding and non-coding variants seem to be relatively accurate (64,65). Owing to the exhaustive preprocessing and statistical fine-mapping steps combined with the necessary data to annotate variants we have yet to see these one-size-fits-all functionally informed gene prioritization methods be made available in user-friendly tools and broadly applicable tools.

Additionally, there are methods for prioritizing genes in non-coding risk loci, such as Polygenic Prioritization Score (PoPS) (66) or the simple nearest gene method, which do not utilize experimentally derived SNP-to-gene data. PoPS is a ridge regression framework that leverages the polygenic signal in coding regions to identify enriched shared features such as pathways, protein–protein interaction networks and co-expression modules and prioritize genes in all risk loci (including non-coding loci). The nearest gene method simply states that the nearest gene body to the (lead) variant is most likely the effector gene. This is underpinned by the recent whole exome sequencing (WES) of 454 787 UK biobank participants which showed that genes containing rare coding variants significant at *P* ≤ 10^−7^ are 45.5 times more likely to be the closest gene to a GWAS lead variant for the same trait than expected by chance (67). Weeks *et al*. demonstrate that overlapping the nearest gene with PoPS prioritizations, or either with any prioritizations based on functional data, the causal gene prediction accuracy is around 80%. Overall, these methods are less interpretable than causal gene prediction based on functional data, yet when combined, they can prioritize causal genes in non-coding risk loci with decent accuracy.

**Table 4:** Commonly used gene prioritization tools and methods for integrating functional with associated variants

Name	Method	Description	Strenghts and weaknesses	Non-coding specific
Experimental SNP-to-gene data	Dependent on dataset	Overlap associated variants with SNP/region to gene datasets derived from functional experiments, such as transcription factor gene pairs or enhancer gene pairs	+ Provides insight into biologically affected mechanism + Provides evidence for functional impact of variant − Time-consuming − Actual causal mechanism might not be captured by current datasets	Mostly
L2G (65)	Supervised learning	Gradient boosting algorithm trained on fine-mapped GWAS loci with a known causal gene. Pulls features from multiple functional experiments	+ Publicly available GWAS often present in the L2G database − Impractical to use owing to large data and computational requirements − Requires fine-mapping	No
cS2G (64)	The weighted linear scoring framework	A regression model that aims to prioritize the causal variant in a fine-mapped locus. Pulls features from several functional locus-to-gene prioritization methods	+ State-of-the-art performance − Requires fine-mapping	No
PoPS (66)	Ridge regression	Regression of binary gene features shared between genes onto gene–trait correlations derived from GWAS hits within gene bodies. Prioritize genes with most shared features within a (non-coding) risk locus	+ Specific to GWAS of interest − Some affinity with Python/command line needed − Prioritization scores not interpretable − For high accuracy prioritizations need to be combined with functional data derived/nearest gene prioritizations	No
Nearest gene prioritization	Select the nearest gene to GWAS lead variant	Select the nearest gene (either gene body or transcription start site) to the non-coding rare/lead variant	+ Simple + Can be done for any signal − Can only be done for one gene per risk locus unless fine-mapped − Non-interpretable − Needs to be combined with another metric to reach high predictive accuracy	No

## Recommendations

**Figure 1:**
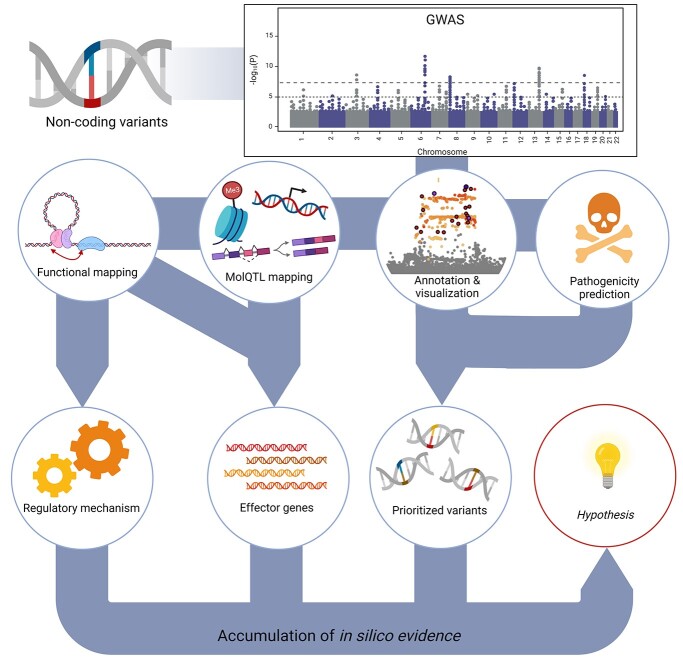
Workflow from non-coding variants to disease insights. Relevant annotation and visualization tools are highlighted in Table 1. Pathogenicity prediction tools are listed in Table 2. Tools and methods for MolQTL analysis are listed in Table 3. Finally, tools and methods for functional mapping and integration of experimental SNP-to-gene data can be found in Table 4.

Throughout this review, we have showcased multiple computational tools for the interpretation of non-coding variants in a disease GWAS context. At its core, *in silico* analysis of GWAS results remains a highly complex task, especially when analyzing non-coding variants. Eventually, a researcher must be able to integrate multiple lines of converging evidence to form a plausible hypothesis of which implicated variants might indeed be causal, and how these variants disrupt normal cell function. Here we provide a proposed workflow that can be used for the post-GWAS analysis of non-coding variants (Fig. 1).

The workflow highlighted before allows for the accumulation of multiple lines of evidence in order to identify putative causal variants and their implications for disease. Eventually no *in silico* evidence is as robust as *in vivo/vitro* evidence. Nevertheless, the *in silico* analysis of disease-associated variants is an invaluable resource for identifying which variants, genes, pathways and cell types should be considered for functional follow-up studies.

## Future developments

The variety of different tools and strategies to interpret non-coding variants in a disease context touched upon in this review highlight one underlying truth. There is currently no perfect, one-size-fits-all tool or method to find causal SNPs from GWAS findings. As the costs of WES and WGS are decreasing it is feasible that rare and deleterious coding variants of large effect size will allow us to more easily pinpoint causal genes for a variety of disorders. This luxury is absent for non-coding variants. Therefore, we foresee the need for continued improvement of non-coding variant analysis tools. Accumulation of massively parallel reporter (42,62) and CRISPR/Cas9 (63) assays may in the coming years be a valuable additional source of data for the improvement of predictive algorithms.

## Conclusions

The number of databases, roadmaps and atlases (3,16–19) that have been developed over the last years make this an exciting time for investigating the complex and diverse regulatory mechanisms in the non-coding part of the genome. There is a broad range of tools available that allow researchers to accumulate evidence for regulatory effects of non-coding variants that are expected to be causally associated with a disease phenotype (Tables 1–4). Finding the correct tool for your research question can be a challenge. Both the prediction of causal/pathogenic variants and the prediction of effector genes and pathways of non-coding variants display a large amount of variation in their performance. More frequent, comprehensive benchmarks would allow researchers to make more informed choices on which tool suits their research best.

Despite differences in performance across tools, the abundance of data allows for the development of hypotheses on disease mechanisms by the accumulation of functional evidence across multiple tracks from non-coding GWAS hits. Currently, there is no one-size-fits-all tool for identifying causal genes and mechanisms from disease-associated non-coding variants. We therefore strongly encourage the continuation of the development of tools that prioritize causal genes and variants, capitalizing on novel data and insights. These tools should be easy to use for researchers without (bio)informatics backgrounds, and provide interpretable prioritization metrics.

In the end, the tools and suggested workflow highlighted in this review are merely instruments for the development of novel insights and hypotheses into the mechanisms driving a specific disease. Although their results can be used as supporting evidence, *in vitro/vivo* experimental validation remains necessary to truly establish a causal relationship between non-coding variants and disease.

## References

[1] Graur, D., Zheng, Y., Price, N., Azevedo, R.B.R., Zufall, R.A. and Elhaik, E. (2013) On the immortality of television sets: “Function” in the human genome according to the evolution-free gospel of ENCODE. Genome Biol. Evol., 5, 578–590. 23431001 10.1093/gbe/evt028 PMC3622293

[2] Doolittle, W.F. (2013) Is junk DNA bunk? A critique of ENCODE. Proc. Natl. Acad. Sci., 110, 5294–5300. 23479647 10.1073/pnas.1221376110 PMC3619371

[3] Dunham, I., Kundaje, A., Aldred, S.F., Collins, P.J., Davis, C.A., Doyle, F., Epstein, C.B., Frietze, S., Harrow, J., Kaul, R. et al. (2012) An integrated encyclopedia of DNA elements in the human genome. Nature, 489, 57–74. 22955616 10.1038/nature11247 PMC3439153

[4] Pennisi, E. (2012) ENCODE project writes eulogy for junk DNA. Science, 337, 1159–1161. 22955811 10.1126/science.337.6099.1159

[5] Barrett, L.W., Fletcher, S. and Wilton, S.D. (2012) Regulation of eukaryotic gene expression by the untranslated gene regions and other non-coding elements. Cell. Mol. Life Sci., 69, 3613–3634. 22538991 10.1007/s00018-012-0990-9 PMC3474909

[6] Claussnitzer, M., Cho, J.H., Collins, R., Cox, N.J., Dermitzakis, E.T., Hurles, M.E., Kathiresan, S., Kenny, E.E., Lindgren, C.M., MacArthur, D.G. et al. (2020) A brief history of human disease genetics. Nature, 577, 179–189. 31915397 10.1038/s41586-019-1879-7 PMC7405896

[7] Watanabe, K., Stringer, S., Frei, O., Umićević Mirkov, M., de Leeuw, C., Polderman, T.J.C., van der Sluis, S., Andreassen, O.A., Neale, B.M. and Posthuma, D. (2019) A global overview of pleiotropy and genetic architecture in complex traits. Nat. Genet., 51, 1339–1348. 31427789 10.1038/s41588-019-0481-0

[8] Bauer, D.E., Kamran, S.C., Lessard, S., Xu, J., Fujiwara, Y., Lin, C., Shao, Z., Canver, M.C., Smith, E.C., Pinello, L. et al. (2013) An erythroid enhancer of BCL11A subject to genetic variation determines fetal hemoglobin level. Science, 342, 253–257. 24115442 10.1126/science.1242088 PMC4018826

[9] Corradin, O. and Scacheri, P.C. (2014) Enhancer variants: evaluating functions in common disease. Genome Med., 6, 85. 25473424 10.1186/s13073-014-0085-3 PMC4254432

[10] Deplancke, B., Alpern, D. and Gardeux, V. (2016) The genetics of transcription factor DNA binding variation. Cell, 166, 538–554. 27471964 10.1016/j.cell.2016.07.012

[11] Kadota, M., Yang, H.H., Hu, N., Wang, C., Hu, Y., Taylor, P.R., Buetow, K.H. and Lee, M.P. (2007) Allele-specific chromatin immunoprecipitation studies show genetic influence on chromatin state in human genome. PLoS Genet., 3, e81. 17511522 10.1371/journal.pgen.0030081 PMC1868950

[12] Heidenreich, B., Rachakonda, P.S., Hemminki, K. and Kumar, R. (2014) TERT promoter mutations in cancer development. Curr. Opin. Genet. Dev., 24, 30–37. 24657534 10.1016/j.gde.2013.11.005

[13] Horn, S., Figl, A., Rachakonda, P.S., Fischer, C., Sucker, A., Gast, A., Kadel, S., Moll, I., Nagore, E., Hemminki, K., Schadendorf, D. and Kumar, R. (2013) TERT promoter mutations in familial and sporadic melanoma. Science, 339, 959–961. 23348503 10.1126/science.1230062

[14] Huang, F.W., Hodis, E., Xu, M.J., Kryukov, G.V., Chin, L. and Garraway, L.A. (2013) highly recurrent TERT promoter mutations in human melanoma. Science, 339, 957–959. 23348506 10.1126/science.1229259 PMC4423787

[15] Killela, P.J., Reitman, Z.J., Jiao, Y., Bettegowda, C., Agrawal, N., Diaz, L.A., Friedman, A.H., Friedman, H., Gallia, G.L., Giovanella, B.C. et al. (2013) TERT promoter mutations occur frequently in gliomas and a subset of tumors derived from cells with low rates of self-renewal. Proc. Natl. Acad. Sci., 110, 6021–6026. 23530248 10.1073/pnas.1303607110 PMC3625331

[16] Davis, C.A., Hitz, B.C., Sloan, C.A., Chan, E.T., Davidson, J.M., Gabdank, I., Hilton, J.A., Jain, K., Baymuradov, U.K., Narayanan, A.K. et al. (2018) The Encyclopedia of DNA elements (ENCODE): data portal update. Nucleic Acids Res., 46, D794–D801. 29126249 10.1093/nar/gkx1081 PMC5753278

[17] Andersson, R., Gebhard, C., Miguel-Escalada, I., Hoof, I., Bornholdt, J., Boyd, M., Chen, Y., Zhao, X., Schmidl, C., Suzuki, T. et al. (2014) An atlas of active enhancers across human cell types and tissues. Nature, 507, 455–461. 24670763 10.1038/nature12787 PMC5215096

[18] Bernstein, B.E., Stamatoyannopoulos, J.A., Costello, J.F., Ren, B., Milosavljevic, A., Meissner, A., Kellis, M., Marra, M.A., Beaudet, A.L., Ecker, J.R. et al. (2010) The NIH roadmap epigenomics mapping consortium. Nat. Biotechnol., 28, 1045–1048. 20944595 10.1038/nbt1010-1045 PMC3607281

[19] Lonsdale, J., Thomas, J., Salvatore, M., Phillips, R., Lo, E., Shad, S., Hasz, R., Walters, G., Garcia, F., Young, N. et al. (2013) The genotype-tissue expression (GTEx) project. Nat. Genet., 45, 580–585. 23715323 10.1038/ng.2653 PMC4010069

[20] Wang, K., Li, M. and Hakonarson, H. (2010) ANNOVAR: functional annotation of genetic variants from high-throughput sequencing data. Nucleic Acids Res., 38, e164. 20601685 10.1093/nar/gkq603 PMC2938201

[21] Watanabe, K., Taskesen, E., van Bochoven, A. and Posthuma, D. (2017) Functional mapping and annotation of genetic associations with FUMA. Nat. Commun., 8, 1826. 29184056 10.1038/s41467-017-01261-5 PMC5705698

[22] Paila, U., Chapman, B.A., Kirchner, R. and Quinlan, A.R. (2013) GEMINI: integrative exploration of genetic variation and genome annotations. PLoS Comput. Biol., 9, e1003153. 23874191 10.1371/journal.pcbi.1003153 PMC3715403

[23] Ward, L.D. and Kellis, M. (2012) HaploReg: a resource for exploring chromatin states, conservation, and regulatory motif alterations within sets of genetically linked variants. Nucleic Acids Res., 40, D930–D934. 22064851 10.1093/nar/gkr917 PMC3245002

[24] Boyle, A.P., Hong, E.L., Hariharan, M., Cheng, Y., Schaub, M.A., Kasowski, M., Karczewski, K.J., Park, J., Hitz, B.C., Weng, S., Cherry, J.M. and Snyder, M. (2012) Annotation of functional variation in personal genomes using RegulomeDB. Genome Res., 22, 1790–1797. 22955989 10.1101/gr.137323.112 PMC3431494

[25] McLaren, W., Gil, L., Hunt, S.E., Riat, H.S., Ritchie, G.R.S., Thormann, A., Flicek, P. and Cunningham, F. (2016) The Ensembl variant effect predictor. Genome Biol., 17, 122. 27268795 10.1186/s13059-016-0974-4 PMC4893825

[26] Boughton, A.P., Welch, R.P., Flickinger, M., VandeHaar, P., Taliun, D., Abecasis, G.R. and Boehnke, M. (2021) LocusZoom.js: interactive and embeddable visualization of genetic association study results. Bioinformatics, 37, 3017–3018. 10.1093/bioinformatics/btab186 PMC8479674 33734315

[27] Claussnitzer, M., Dankel, S.N., Kim, K.-H., Quon, G., Meuleman, W., Haugen, C., Glunk, V., Sousa, I.S., Beaudry, J.L., Puviindran, V. et al. (2015) FTO obesity variant circuitry and adipocyte browning in humans. N. Engl. J. Med., 373, 895–907. 26287746 10.1056/NEJMoa1502214 PMC4959911

[28] Huang, Y.-F., Gulko, B. and Siepel, A. (2017) Fast, scalable prediction of deleterious noncoding variants from functional and population genomic data. Nat. Genet., 49, 618–624. 28288115 10.1038/ng.3810 PMC5395419

[29] Ionita-Laza, I., McCallum, K., Xu, B. and Buxbaum, J.D. (2016) A spectral approach integrating functional genomic annotations for coding and noncoding variants. Nat. Genet., 48, 214–220. 26727659 10.1038/ng.3477 PMC4731313

[30] Ritchie, G.R.S., Dunham, I., Zeggini, E. and Flicek, P. (2014) Functional annotation of noncoding sequence variants. Nat. Methods, 11, 294–296. 24487584 10.1038/nmeth.2832 PMC5015703

[31] Shihab, H.A., Rogers, M.F., Gough, J., Mort, M., Cooper, D.N., Day, I.N.M., Gaunt, T.R. and Campbell, C. (2015) An integrative approach to predicting the functional effects of non-coding and coding sequence variation. Bioinformatics, 31, 1536–1543. 25583119 10.1093/bioinformatics/btv009 PMC4426838

[32] Rogers, M.F., Shihab, H.A., Mort, M., Cooper, D.N., Gaunt, T.R. and Campbell, C. (2018) FATHMM-XF: accurate prediction of pathogenic point mutations via extended features. Bioinformatics, 34, 511–513. 28968714 10.1093/bioinformatics/btx536 PMC5860356

[33] Chen, L., Jin, P. and Qin, Z.S. (2016) DIVAN: accurate identification of non-coding disease-specific risk variants using multi-omics profiles. Genome Biol., 17, 252. 27923386 10.1186/s13059-016-1112-z PMC5139035

[34] Fu, Y., Liu, Z., Lou, S., Bedford, J., Mu, X.J., Yip, K.Y., Khurana, E. and Gerstein, M. (2014) FunSeq2: a framework for prioritizing noncoding regulatory variants in cancer. Genome Biol., 15, 480. 25273974 10.1186/s13059-014-0480-5 PMC4203974

[35] Kircher, M., Witten, D.M., Jain, P., O’Roak, B.J., Cooper, G.M. and Shendure, J. (2014) A general framework for estimating the relative pathogenicity of human genetic variants. Nat. Genet., 46, 310–315. 24487276 10.1038/ng.2892 PMC3992975

[36] Quang, D., Chen, Y. and Xie, X. (2015) DANN: a deep learning approach for annotating the pathogenicity of genetic variants. Bioinformatics, 31, 761–763. 25338716 10.1093/bioinformatics/btu703 PMC4341060

[37] Vitsios, D., Dhindsa, R.S., Middleton, L., Gussow, A.B. and Petrovski, S. (2021) Prioritizing non-coding regions based on human genomic constraint and sequence context with deep learning. Nat. Commun., 12, 1504. 33686085 10.1038/s41467-021-21790-4 PMC7940646

[38] Zhou, J. and Troyanskaya, O.G. (2015) Predicting effects of noncoding variants with deep learning-based sequence model. Nat. Methods, 12, 931–934. 26301843 10.1038/nmeth.3547 PMC4768299

[39] Lee, D., Gorkin, D.U., Baker, M., Strober, B.J., Asoni, A.L., McCallion, A.S. and Beer, M.A. (2015) A method to predict the impact of regulatory variants from DNA sequence. Nat. Genet., 47, 955–961. 26075791 10.1038/ng.3331 PMC4520745

[40] Wang, Z., Zhao, G., Li, B., Fang, Z., Chen, Q., Wang, X., Luo, T., Wang, Y., Zhou, Q., Li, K. et al. (2022) Performance comparison of computational methods for the prediction of the function and pathogenicity of non-coding variants. Genom. Proteom. Bioinform.10.1016/j.gpb.2022.02.00235272052

[41] Drubay, D., Gautheret, D. and Michiels, S. (2018) A benchmark study of scoring methods for non-coding mutations. Bioinformatics, 34, 1635–1641. 29340599 10.1093/bioinformatics/bty008

[42] Cooper, Y.A., Davis, J.E., Kosuri, S., Coppola, G. and Geschwind, D.H. (2021) Functional regulatory variants implicate distinct transcriptional networks in dementia. Functional regulatory variants implicate distinct transcriptional networks in dementia. BioRxiv, 2021.06.14.448395.

[43] Chalmers, Z.R., Connelly, C.F., Fabrizio, D., Gay, L., Ali, S.M., Ennis, R., Schrock, A., Campbell, B., Shlien, A., Chmielecki, J. et al. (2017) Analysis of 100,000 human cancer genomes reveals the landscape of tumor mutational burden. Genome Med., 9, 1–14. 28420421 10.1186/s13073-017-0424-2 PMC5395719

[44] Park, J.S., Lee, J., Jung, E.S., Kim, M.-H., Kim, I.B., Son, H., Kim, S., Kim, S., Park, Y.M., Mook-Jung, I., Yu, S.J. and Lee, J.H. (2019) Brain somatic mutations observed in Alzheimer’s disease associated with aging and dysregulation of tau phosphorylation. Nat. Commun., 10, 3090. 31300647 10.1038/s41467-019-11000-7 PMC6626023

[45] Wallis, Y., Payne, S., McAnulty, C., Bodmer, D. and Sister, E. (2013) Practice guidelines for the evaluation of pathogenicity and the reporting of sequence variants in clinical molecular. Genetics, Association for Clinical Genetic Science and the Dutch Society of Clinical Genetic Laboratory Specialists.

[46] Giambartolomei, C., Vukcevic, D., Schadt, E.E., Franke, L., Hingorani, A.D., Wallace, C. and Plagnol, V. (2014) Bayesian test for colocalisation between pairs of genetic association studies using summary statistics. PLoS Genet., 10, e1004383. 24830394 10.1371/journal.pgen.1004383 PMC4022491

[47] Zhang, T., Klein, A., Sang, J., Choi, J. and Brown, K.M. (2022) ezQTL: a web platform for interactive visualization and colocalization of quantitative trait loci and GWAS. BioRxiv, 2022.03.08.483491 10.1016/j.gpb.2022.05.004 PMC9801033 35643189

[48] Ioannidis, N.M., Davis, J.R., DeGorter, M.K., Larson, N.B., McDonnell, S.K., French, A.J., Battle, A.J., Hastie, T.J., Thibodeau, S.N., Montgomery, S.B. et al. (2017) FIRE: functional inference of genetic variants that regulate gene expression. Bioinformatics, 33, 3895–3901. 28961785 10.1093/bioinformatics/btx534 PMC5860093

[49] Chen, L., Wang, Y., Yao, B., Mitra, A., Wang, X. and Qin, X. (2019) TIVAN: tissue-specific cis-eQTL single nucleotide variant annotation and prediction. Bioinformatics, 35, 1573–1575. 30304335 10.1093/bioinformatics/bty872

[50] Westra, H.-J., Peters, M.J., Esko, T., Yaghootkar, H., Schurmann, C., Kettunen, J., Christiansen, M.W., Fairfax, B.P., Schramm, K., Powell, J.E. et al. (2013) Systematic identification of trans eQTLs as putative drivers of known disease associations. Nat. Genet., 45, 1238–1243. 24013639 10.1038/ng.2756 PMC3991562

[51] Fromer, M., Roussos, P., Sieberts, S.K., Johnson, J.S., Kavanagh, D.H., Perumal, T.M., Ruderfer, D.M., Oh, E.C., Topol, A., Shah, H.R. et al. (2016) Gene expression elucidates functional impact of polygenic risk for schizophrenia. Nat. Neurosci., 19, 1442–1453. 27668389 10.1038/nn.4399 PMC5083142

[52] Won, H., de la Torre-Ubieta, L., Stein, J.L., Parikshak, N.N., Huang, J., Opland, C.K., Gandal, M.J., Sutton, G.J., Hormozdiari, F., Lu, D. et al. (2016) Chromosome conformation elucidates regulatory relationships in developing human brain. Nature, 538, 523–527. 27760116 10.1038/nature19847 PMC5358922

[53] Guo, H., Fortune, M.D., Burren, O.S., Schofield, E., Todd, J.A. and Wallace, C. (2015) Integration of disease association and eQTL data using a Bayesian colocalisation approach highlights six candidate causal genes in immune-mediated diseases. Hum. Mol. Genet., 24, 3305–3313. 25743184 10.1093/hmg/ddv077 PMC4498151

[54] Mostafavi, H., Spence, J.P., Naqvi, S. and Pritchard, J.K. (2022) Limited overlap of eQTLs and GWAS hits due to systematic differences in discovery. BioRxiv, 2022.05.07.491045.

[55] Jung, I., Schmitt, A., Diao, Y., Lee, A.J., Liu, T., Yang, D., Tan, C., Eom, J., Chan, M., Chee, S. et al. (2019) A compendium of promoter-centered long-range chromatin interactions in the human genome. Nat. Genet., 51, 1442–1449. 31501517 10.1038/s41588-019-0494-8 PMC6778519

[56] Javierre, B.M., Burren, O.S., Wilder, S.P., Kreuzhuber, R., Hill, S.M., Sewitz, S., Cairns, J., Wingett, S.W., Várnai, C., Thiecke, M.J. et al. (2016) Lineage-specific genome architecture links enhancers and non-coding disease variants to target gene promoters. Cell, 167, 1369–1384.e19. 27863249 10.1016/j.cell.2016.09.037 PMC5123897

[57] Nasser, J., Bergman, D.T., Fulco, C.P., Guckelberger, P., Doughty, B.R., Patwardhan, T.A., Jones, T.R., Nguyen, T.H., Ulirsch, J.C., Lekschas, F. et al. (2021) Genome-wide enhancer maps link risk variants to disease genes. Nature, 593, 238–243. 33828297 10.1038/s41586-021-03446-x PMC9153265

[58] Pliner, H.A., Packer, J.S., McFaline-Figueroa, J.L., Cusanovich, D.A., Daza, R.M., Aghamirzaie, D., Srivatsan, S., Qiu, X., Jackson, D., Minkina, A. et al. (2018) Cicero predicts cis-regulatory DNA interactions from single-cell chromatin accessibility data. Mol. Cell, 71, 858–871.e8. 30078726 10.1016/j.molcel.2018.06.044 PMC6582963

[59] Satpathy, A.T., Granja, J.M., Yost, K.E., Qi, Y., Meschi, F., McDermott, G.P., Olsen, B.N., Mumbach, M.R., Pierce, S.E., Corces, M.R. et al. (2019) Massively parallel single-cell chromatin landscapes of human immune cell development and intratumoral T cell exhaustion. Nat. Biotechnol., 37, 925–936. 31375813 10.1038/s41587-019-0206-z PMC7299161

[60] Thurman, R.E., Rynes, E., Humbert, R., Vierstra, J., Maurano, M.T., Haugen, E., Sheffield, N.C., Stergachis, A.B., Wang, H., Vernot, B. et al. (2012) The accessible chromatin landscape of the human genome. Nature, 489, 75–82. 22955617 10.1038/nature11232 PMC3721348

[61] Fulco, C.P., Nasser, J., Jones, T.R., Munson, G., Bergman, D.T., Subramanian, V., Grossman, S.R., Anyoha, R., Doughty, B.R., Patwardhan, T.A. et al. (2019) Activity-by-contact model of enhancer–promoter regulation from thousands of CRISPR perturbations. Nat. Genet., 51, 1664–1669. 31784727 10.1038/s41588-019-0538-0 PMC6886585

[62] Tewhey, R., Kotliar, D., Park, D.S., Liu, B., Winnicki, S., Reilly, S.K., Andersen, K.G., Mikkelsen, T.S., Lander, E.S., Schaffner, S.F. and Sabeti, P.C. (2016) Direct identification of hundreds of expression-modulating variants using a multiplexed reporter assay. Cell, 165, 1519–1529. 27259153 10.1016/j.cell.2016.04.027 PMC4957403

[63] Gasperini, M., Findlay, G.M., McKenna, A., Milbank, J.H., Lee, C., Zhang, M.D., Cusanovich, D.A. and Shendure, J. (2017) CRISPR/Cas9-mediated scanning for regulatory elements required for HPRT1 expression via thousands of large, programmed genomic deletions. Am. J. Hum. Genet., 101, 192–205. 28712454 10.1016/j.ajhg.2017.06.010 PMC5544381

[64] Gazal, S., Weissbrod, O., Hormozdiari, F., Dey, K.K., Nasser, J., Jagadeesh, K.A., Weiner, D.J., Shi, H., Fulco, C.P., O’Connor, L.J. et al. (2022) Combining SNP-to-gene linking strategies to identify disease genes and assess disease omnigenicity. Nat. Genet., 54, 827–836. 35668300 10.1038/s41588-022-01087-y PMC9894581

[65] Mountjoy, E., Schmidt, E.M., Carmona, M., Schwartzentruber, J., Peat, G., Miranda, A., Fumis, L., Hayhurst, J., Buniello, A., Karim, M.A. et al. (2021) An open approach to systematically prioritize causal variants and genes at all published human GWAS trait-associated loci. Nat. Genet., 53, 1527–1533. 34711957 10.1038/s41588-021-00945-5 PMC7611956

[66] Weeks, E.M., Ulirsch, J.C., Cheng, N.Y., Trippe, B.L., Fine, R.S., Miao, J., Patwardhan, T.A., Kanai, M., Nasser, J., Fulco, C.P. et al. (2020) Leveraging polygenic enrichments of gene features to predict genes underlying complex traits and diseases. MedRxiv, 2020.09.08.20190561. 10.1038/s41588-023-01443-6 PMC10836580 37443254

[67] Backman, J.D., Li, A.H., Marcketta, A., Sun, D., Mbatchou, J., Kessler, M.D., Benner, C., Liu, D., Locke, A.E., Balasubramanian, S. et al. (2021) Exome sequencing and analysis of 454,787 UK Biobank participants. Nature, 599, 628–634. 34662886 10.1038/s41586-021-04103-z PMC8596853

[68] Otlu, B., Firtina, C., Keleş, S. and Tastan, O. (2017) GLANET: genomic loci annotation and enrichment tool. Bioinformatics, 33, 2818–2828. 28541490 10.1093/bioinformatics/btx326 PMC6355098

[69] Jäger, M., Wang, K., Bauer, S., Smedley, D., Krawitz, P. and Robinson, P.N. (2014) Jannovar: a Java Library for exome annotation. Hum. Mutat., 35, 548–555. 24677618 10.1002/humu.22531

[70] Cingolani, P., Platts, A., Wang, L.L., Coon, M., Nguyen, T., Wang, L., Land, S.J., Lu, X. and Ruden, D.M. (2012) A program for annotating and predicting the effects of single nucleotide polymorphisms, SnpEff. Fly (Austin), 6, 80–92. 22728672 10.4161/fly.19695 PMC3679285

[71] Pan, Q., Liu, Y.-J., Bai, X.-F., Han, X.-L., Jiang, Y., Ai, B., Shi, S.-S., Wang, F., Xu, M.-C., Wang, Y.-Z. et al. (2021) VARAdb: a comprehensive variation annotation database for human. Nucleic Acids Res., 49, D1431–D1444. 33095866 10.1093/nar/gkaa922 PMC7779011

[72] Gao, L., Uzun, Y., Gao, P., He, B., Ma, X., Wang, J., Han, S. and Tan, K. (2018) Identifying noncoding risk variants using disease-relevant gene regulatory networks. Nat. Commun., 9, 702. 29453388 10.1038/s41467-018-03133-y PMC5816022

[73] Hormozdiari, F., van de Bunt, M., Segrè, A.V., Li, X., Joo, J.W.J., Bilow, M., Sul, J.H., Sankararaman, S., Pasaniuc, B. and Eskin, E. (2016) Colocalization of GWAS and eQTL signals detects target genes. Am. J. Hum. Genet., 99, 1245–1260. 27866706 10.1016/j.ajhg.2016.10.003 PMC5142122

[74] Wen, X., Pique-Regi, R. and Luca, F. (2017) Integrating molecular QTL data into genome-wide genetic association analysis: probabilistic assessment of enrichment and colocalization. PLoS Genet., 13, e1006646. 28278150 10.1371/journal.pgen.1006646 PMC5363995

[75] Liu, B., Gloudemans, M.J., Rao, A.S., Ingelsson, E. and Montgomery, S.B. (2019) Abundant associations with gene expression complicate GWAS follow-up. Nat. Genet., 51, 768–769. 31043754 10.1038/s41588-019-0404-0 PMC6904208

